# The role of human milk nutrients in preventing necrotizing enterocolitis

**DOI:** 10.3389/fped.2023.1188050

**Published:** 2023-06-02

**Authors:** Ahmad S. Sami, Lauren C. Frazer, Claire M. Miller, Dhirendra K. Singh, Lynda G. Clodfelter, Kelly A. Orgel, Misty Good

**Affiliations:** ^1^ Division of Pediatric Gastroenterology, Department of Pediatrics, University of North Carolina at Chapel Hill, Chapel Hill, NC, United States; ^2^ Division of Neonatal-Perinatal Medicine, Department of Pediatrics, University of North Carolina at Chapel Hill, Chapel Hill, NC, United States

**Keywords:** breast milk, neonates, prematurity, necrotizing enterocolitis (NEC), intestine, nutrients

## Abstract

Necrotizing enterocolitis (NEC) is an intestinal disease that primarily impacts preterm infants. The pathophysiology of NEC involves a complex interplay of factors that result in a deleterious immune response, injury to the intestinal mucosa, and in its most severe form, irreversible intestinal necrosis. Treatments for NEC remain limited, but one of the most effective preventative strategies for NEC is the provision of breast milk feeds. In this review, we discuss mechanisms by which bioactive nutrients in breast milk impact neonatal intestinal physiology and the development of NEC. We also review experimental models of NEC that have been used to study the role of breast milk components in disease pathophysiology. These models are necessary to accelerate mechanistic research and improve outcomes for neonates with NEC.

## Introduction

Necrotizing enterocolitis (NEC) is a severe gastrointestinal disease that impacts 2%–7% of preterm infants (1). Risk factors for NEC include prematurity, low birth weight, delivery via cesarean section, lack of breast milk feeds, microbial dysbiosis, inadequate intestinal perfusion, and exposure to medications such as antibiotics and acid blockers (2). Disease pathogenesis is characterized by unrestrained inflammation, injury to the intestinal epithelium, and bowel ischemia, which can rapidly progress to bowel necrosis, sepsis, and death (3). Treatment options for NEC include the discontinuation of enteral nutrition, gastric decompression, broad-spectrum antibiotics, and surgical removal of necrotic bowel (3). There are no targeted therapies available due to our incomplete understanding of disease pathogenesis; however, it has been well described that breast milk feedings are a protective factor against the development of NEC (4–7). Bioactive components in human milk have been demonstrated to reduce intestinal inflammation, enhance stem cell proliferation, decrease enterocyte apoptosis, and promote the development of a healthy microbiome (5–11).

**Figure 1:**
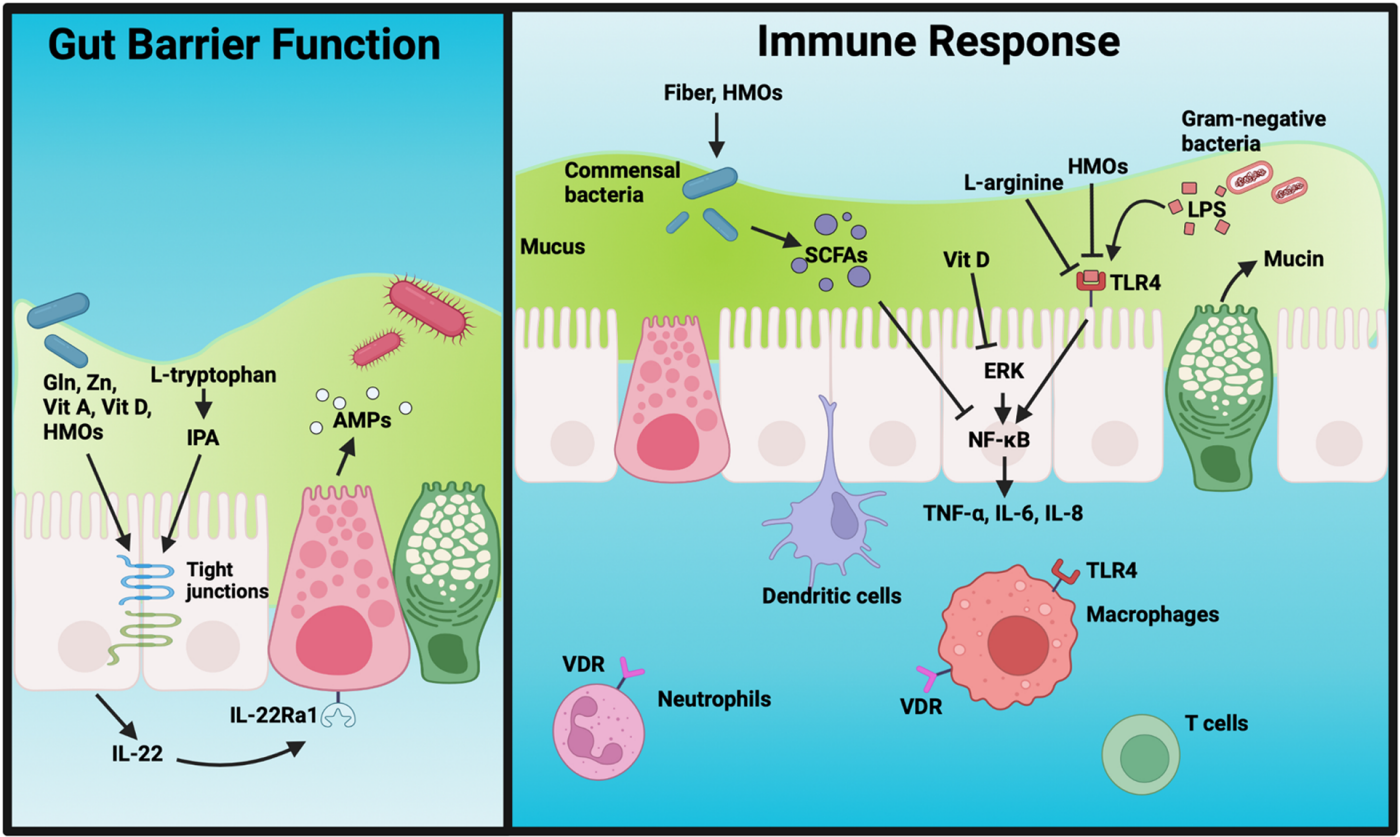
Summary of the impact of nutritional factors on gut barrier integrity and the mucosal immune response. Nutritional components improve the intestinal barrier by enhancing the expression of tight junctions, increasing IL-22 production, promoting mucus secretion, and inducing Paneth cell AMP release. They also have diverse effects on the immune response via modulation of the microbiome, downregulation of inflammatory signaling pathways, and prevention of potentially deleterious immune cell activation. Gln, glutamine; Zn, zinc; Vit A, vitamin A; Vit D, vitamin D; HMOs, human milk oligosaccharides; IPA, 3-indole propionic acid; AMP, antimicrobial peptides; SCFAs, short-chain fatty acids; LPS, lipopolysaccharide; VDR, vitamin D receptor; TLR4, toll-like receptor 4; ERK, extracellular signal-regulated protein kinase; ROS, reactive oxygen species. Figure created with Biorender.com.

In this review, we discuss important components of breast milk and their role in intestinal immune homeostasis, barrier function, and the prevention of NEC (Figure 1). Finally, we outline models of NEC that can be utilized for mechanistic studies into the impact of breast milk components on intestinal physiology.

## Lipids

Breast milk lipids are important in supporting a diverse array of physiologic functions in early life, such as organogenesis, lipid membrane development, and signaling molecule synthesis (12). Long-chain polyunsaturated fatty acids (LC-PUFAs) are a class of bioactive lipids that are predominately acquired during the third trimester of pregnancy (13). This translates into inadequate LC-PUFA stores in preterm neonates and rapid declines in LC-PUFA levels after birth (14). The impact of these deficiencies on intestinal health remains an area of active research. In a study of preterm piglets, enteral provision of a lipid emulsion containing varying ratios of the LC-PUFAs arachidonic acid (ARA, C20:4n-6) and docosahexaenoic acid (DHA, 22:6n-3) found greater villus height in the ileum of piglets that were adequately supplemented with ARA (15). In a rat model of NEC, supplementation of formula with ARA and DHA led to reduced disease severity relative to controls (16). Finally, *in vitro* studies using human fetal intestinal epithelial cells found that treatment with ARA and/or DHA reduced cytokine production in response to an inflammatory stimulus (17). Additional research is needed in the form of both preclinical models and clinical trials to determine the optimal dose and ratio of LC-PUFA supplementation to support intestinal development and reduce the risk of NEC in preterm infants.

## Lactoferrin

Lactoferrin is an abundant component of the whey protein fraction of breast milk that has a diverse array of potentially beneficial functions, including enhancing immunity, controlling inflammation, and promoting intestinal epithelial cell growth (18–21). Host defense properties of lactoferrin arise from iron binding properties as well as direct interactions with microbes and immune cells (22). Clinical trials and a 2020 Cochrane Review have thus far not detected a significant benefit for lactoferrin supplementation in the risk of NEC or mortality for preterm neonates (23–25). Additional studies, such as the Lactoferrin Infant Feeding Trial (LIFT_Canada), are needed to examine the impact of lactoferrin supplementation on the health of preterm neonates (26).

## Human milk oligosaccharides (HMOs)

Human milk oligosaccharides (HMOs) are a family of over 150 structurally complex glycans that are abundant in human milk, with concentrations varying based on the stage of lactation (27–30). HMOs are metabolized by intestinal bacteria such as *Bifidobacteria* and *Lactobacilli* spp., and thus shape the development of the intestinal microbiome (31). Additionally, HMOs serve a diverse array of potentially beneficial roles in the intestine, including augmenting host defense, modulating immune cell function, and enhancing intestinal barrier integrity (32–34). For example, HMOs act as soluble adhesion receptor decoys, blocking the attachment of viral and bacterial pathogens to intestinal epithelial cells (35, 36). HMOs also possess bacteriostatic and bactericidal properties and can modulate intestinal inflammatory responses (34). In addition, maternal breast milk HMO levels have been associated with an infant's risk of developing NEC (37).

The role of HMOs in attenuating inflammatory immune responses in the gut is well described in preclinical models. In a recent study by Suligoj et al., the effects of HMOs on intestinal barrier function were explored using Caco-2 cell monolayers (38). A combination of 2'-O-fucosyllactose (2’FL), the most abundant oligosaccharide in human milk, and lacto-N-neotetraose (LNnT) was shown to significantly decrease paracellular permeability while increasing tight junction protein (claudin-8) expression (38). In an *ex vivo* model of human intestinal tissue, galactosyloligosaccharides (GOs) were shown to downregulate TNF-α and interleukin (IL)-1β production (39). In addition, colostrum HMOs, particularly GOs, attenuated Toll-like receptor (TLR) 3 and TLR5 signaling (32). Lastly, the HMO α-3 sialyllactose was shown to downregulate the expression of the inflammatory cytokines IL-8 and IL-12 in Caco-2 cells by inhibiting nuclear factor-κB (NF-κB) signaling and stimulating peroxisome proliferator-activated receptor gamma (PPAR-γ) expression (40).

Similar anti-inflammatory properties of HMOs have been described in animal models of NEC. For example, in a rat model of NEC, the HMO disialyllacto-N-tetraose (DSLNT) increased survival rates from 73.1% to 95% (*P *< 0.001) and led to a reduction in intestinal pathology (41). A human study found that significantly decreased levels DSLNT in maternal breastmilk were detectable for infants who developed NEC relative to controls (42). In addition, in a mouse model of NEC, administration of 2'FL resulted in a decreased severity of intestinal injury that was associated with improved intestinal perfusion (43). Lastly, the HMOs 2'FL and 6'-sialyllactose (6'-SL) decreased intestinal injury in mouse and piglet models of NEC, which was associated with reductions in TLR4 activation (44). These findings support further investigation into the role of HMO supplementation in the development of a healthy microbiome and prevention of NEC in preterm neonates.

## Dietary amino acids

Dietary amino acids (AA) are a primary energy source for intestinal epithelial cells (45). AA in human milk are predominantly protein-bound, with approximately 5%–10% present in free form (46). Free AA are more readily absorbed into the intestinal circulation than their protein-bound counterparts and contribute significantly to the initial rise in AA serum levels in infants following a feed (47). These free AA support intestinal health and may contribute to preventing NEC in preterm infants (45, 48–51). We will discuss amino acids that have been studied in relationship to NEC.

### Glutamine

Glutamine (Gln) is the most abundant essential free AA in human milk, particularly in the first three months of lactation (52), and a deficiency in circulating Gln is associated with an increased risk of NEC in neonates (53). The beneficial effects of Gln include promoting intestinal epithelial growth, improving barrier function, reducing oxidative stress, and downregulating inflammation.

Gln promotes intestinal growth by providing energy for intestinal epithelial cell proliferation as well as regulating signaling pathways, including the mammalian target of rapamycin (mTOR), mitogen-activated protein kinase (MAPK), and extracellular signal-regulated protein kinase (ERK) pathways (54). Additionally, Gln enhances the effects of growth factors such as epidermal growth factor (EGF), transforming growth factor alpha (TGF*α*), and insulin-like growth factor-1 (IGF-1) (54).

Gln is critical in preventing epithelial cell atrophy in catabolic states and improves barrier function by regulating the expression of tight junction proteins, including claudin-1, occludin, and zonula occludens (ZO-1) (55, 56). In a randomized clinical trial, improved intestinal barrier integrity was observed for preterm neonates receiving enteral Gln (57).

Gln exerts anti-oxidative properties by acting as a substrate for glutathione (GSH) biosynthesis (58). GSH is a tripeptide composed of Gln, glycine, and cysteine that scavenges potentially damaging reactive oxidants and free radicals (58). In a study involving breastfed newborn rats, enteral Gln supplementation reduced markers of oxidative stress in intestinal tissue (59). In another study examining intestinal epithelial cells (IECs) in the setting of oxidative and non-oxidative stress, Gln exerted anti-apoptotic properties by decreasing the level of cleaved caspase-3 and increasing the expression of heat shock proteins (53).

Gln has also been shown to downregulate inflammation. In an *in vitro* study using healthy human intestinal tissue, Gln supplementation downregulated the production of the inflammatory cytokine interleukin-1 beta and upregulated the level of the anti-inflammatory cytokines IL-4 and IL-10 (60). In a rat model of NEC, Gln supplementation was associated with decreased mucosal injury, reduced inflammation, and downregulated expression of the innate immune receptors Toll-like receptor-2 and TLR4 in ileal and colonic tissue (61). Although these studies indicate that Gln may have a beneficial role in intestinal health, a 2016 Cochrane review found that glutamine supplementation was unlikely to significantly improve outcomes for preterm neonates (62).

### L-arginine

L-arginine is a semi-essential amino acid exclusively synthesized by intestinal epithelial cells (63). It is a substrate for nitric oxide (NO) production via the arginine-nitric oxide synthase (NOS) pathway, which plays a vital role in regulating intestinal blood flow and maintaining intestinal integrity (64–67).

The role of L-arginine in NEC has been examined in animal models. In a neonatal piglet model of NEC, reduced arginine levels were detected for preterm piglets prior to NEC onset (68). In addition, supplementation of L-arginine attenuated intestinal injury in another study using this model (69). Mechanistically, this was attributed to enhanced NOS activity and NO production in the intestine (69). In a murine model of NEC, endothelial cell TLR4 activation was associated with increased tissue damage and reduced endothelial NOS (eNOS) activity (70). NEC severity was also found to be increased in eNOS-deficient mice (70). In addition, enteral L-arginine supplementation attenuated hypoxia-reoxygenation-induced bowel injury in a murine model of NEC (71).

In neonates, low levels of circulating L-arginine have been associated with an increased risk of NEC (72). Data from animal studies and RCTs support a potential role for L-arginine supplementation in NEC prevention (59, 63, 68, 69, 71–73). However, a 2017 Cochrane review determined that L-arginine supplementation was associated with a significant reduction in the risk of Bell's Stage 1 but not Stage 2 or 3 NEC (74). A large high-quality study is needed before the routine arginine supplementation for preterm neonates can be implemented.

### L-Tryptophan

L-tryptophan is an essential amino acid found in human milk (75). It is metabolized by tryptophanase expressed by the gut microbiota leading to the production of tryptamine and indole derivatives such as 3-indole propionic acid (IPA) (76). IPA and other tryptophan metabolites have important roles in gut immunity and intestinal barrier integrity.

IPA regulates intestinal barrier function and inflammation by activating the xenobiotic sensor pregnane-X receptor (PXR) (77). PXR activation upregulates the expression of tight junction proteins and downregulates the expression of the inflammatory cytokine tumor necrosis factor-alpha (TNF-α) (78). In epithelial cell-specific PXR-deficient mouse models, enhanced TLR4 signaling results in significant inflammation and loss of intestinal barrier integrity (79).

Indole derivatives also activate the aryl hydrocarbon receptor (AhR) (80, 81). Decreased AhR expression has been associated with the development of NEC, with reduced levels detected in the intestine of neonates, mice, and piglets with NEC (82). Recent evidence from a murine model of NEC found that administration of the AhR proligand indole-3-carbinol (I3C) resulted in reduced severity of NEC (81). Mechanistically, this was associated with downregulated expression of inflammatory cytokines and increased expression of the polyfunctional cytokine IL-22, which has been shown to be an effective therapeutic against NEC (27, 81, 82). Further investigation is needed to determine the protective mechanisms induced by tryptophan metabolites in both animal models and human studies.

## Vitamins

### Vitamin D

Vitamin D is important in immunoregulation and enhancement of intestinal barrier function. Vitamin D exerts diverse immunomodulatory effects by binding to vitamin D receptors (VDR) expressed on immune cells (83, 84). For example, vitamin D inhibits Th17 differentiation and decreases IL-17 production (85). VDR activation also inhibits IL-17 expression in the intestine and reduces IEC apoptosis by blocking NF-κB activation (86). Moreover, activation of VDR signaling reduces tissue damage by promoting T-cell differentiation into Th2 cells rather than inflammatory Th1 cells (87). T-cell phenotype is important in the pathogenesis of NEC, with a role for increased Th17 cells and IL-17-related inflammatory signaling in disease development (88, 89).

Vitamin D deficiency is prevalent in preterm infants, particularly in those below 32 weeks of gestation, and decreased levels of vitamin D have been associated with NEC (90). The role of Vitamin D in supporting intestinal health has been supported by findings in animal models. In a rat model of NEC, vitamin D downregulated TLR4 expression and attenuated apoptosis of intestinal epithelial cells (91). Moreover, vitamin D protected against intestinal barrier disruption and the loss of tight junction proteins by increasing occludin expression (91). In another study, supplementation of vitamin D to lipopolysaccharide (LPS)-treated cells improved cell viability, increased proliferation and growth, and decreased expression of IL-6, IL-1β, and TNF-α (92). Although the protective role of vitamin D is documented using human cell lines and mouse models, there is limited data available on the impact of vitamin D supplementation in NEC prevention.

### Vitamin A

Vitamin A is present in human milk, but concentrations are significantly lower in milk from mothers of preterm infants (93). Vitamin A levels also vary by lactational stage with higher levels found in colostrum relative to mature milk (94). In addition, serum levels of vitamin A in patients with NEC are decreased relative to healthy controls (95). It is possible that Vitamin A is involved in improving intestinal health in preterm neonates, as it has been previously implicated in regulating intestinal immunity and in maintaining intestinal barrier function (96).

Studies in mice found that the intestinal mucosa of vitamin A deficient mice contains a reduced number of immune cells, including macrophages, B- and T-cells (97, 98). Vitamin A deficiency in rats is associated with an increased abundance of *Escherichia coli*, decreased mucin-2 (MUC2) and defensin-6, and upregulation of TLR2 and TLR5 expression in the intestine (99). In a study using a mouse model of NEC, vitamin A supplementation reduced TNF-α and IL-6 mRNA levels relative to controls (100). Vitamin A supplementation also increased the expression levels of claudin-1, occludin, and ZO-1, indicating vitamin A's role in improving intestinal barrier function (95). In another study using murine epithelial cells cultured with retinoic acid (RA), the expression of several tight junction proteins, including occludin, claudin-6, and ZO-1 were induced (101). Finally, decreased permeability and increased transepithelial electrical resistance were noted in another study using intestinal epithelial monolayers grown with all-trans RA (102). These findings support the role of vitamin A in supporting intestinal homeostasis.

## Trace elements

Trace elements are micronutrients present in variable concentrations in human milk (103). Essential trace elements such as zinc (Zn), selenium (Se), and calcium (Ca) improve intestinal barrier integrity, modulate the immune response, and interact with the gut microbiota (104–106).

### Zinc

Zinc (Zn) is involved in essential metabolic functions such as immunoregulation, reduction of oxidative stress, and development of the intestinal tract (107, 108). Zn is primarily acquired in the third trimester of pregnancy leading to low stores in preterm infants (100). Zn content in human milk is dependent on the stage of lactation, while absorption is correlated with the maturity of the infant's gut and bioavailability (109–112).

Zn plays an important role in maintaining intestinal barrier integrity. In a study using Caco-2 cells, induced Zn deficiency led to increased intestinal epithelial permeability and decreased expression of tight junction proteins (113). Similarly, Zn depletion led to the downregulation of occludin and claudin-3 in another study using intestinal Caco-2 cells and *ex vivo* mouse colons (104). Zn has also been shown to directly enhance the production of intestinal epithelial cells in crypts and promote IEC differentiation, particularly in disease states with increased mucosal turnover (110, 114, 115). Lastly, Zn deficiency decreases mucin synthesis through disturbances in the goblet cell homeostasis (116). Taken together, these data suggest the importance of Zn in maintaining intestinal barrier function.

Several studies highlight Zn's regulation of intestinal immune function. In an *in vitro* study using chicken intestinal tissue, Zn supplementation (Zn-Gly) increased the expression of secretory immunoglobulin A (IgA), promoted a Th1 and Th2 balance, and reduced the expression of inflammatory cytokines such as TNF-α and IL-1β (117). Zn is also critical for the normal function and morphology of Paneth cells in animal models (118). Similarly, decreased Paneth cell function occurs in human intestinal tissue in response to low levels of Zn (119).

In addition to its immunomodulatory effects, Zn directly affects the composition of the gut microbiota (120). Zn deficiency reduces gut microbial diversity by indirectly promoting the growth of bacteria adapted to low Zn environments, such as Proteobacteria spp. (120). Several studies have associated Gammaproteobacteria, a class of Proteobacteria, with an increased risk for NEC (121–123). Conversely, Zn excess may also lead to gut dysbiosis. Excess levels of Zn in mice colonized with *Clostridium difficile* were found to exacerbate inflammation and intestinal disease by increasing toxin activity (124). Understanding the interplay between Zn deficiency and the intestinal microbiome could provide new insights into NEC pathophysiology.

## Interaction between nutrients and the gut microbiota in NEC

One of the central roles of human breast milk feeds in neonatal health is shaping the development of the neonatal microbiome. Breast milk contains its own microbiome, and these bacteria directly colonize the neonatal intestine (125, 126). In addition, breast milk components directly influence the composition of the gut microbiome. For example, HMOs can facilitate *Bifidobacteria* and *Lactobacilli* spp. growth (31), and breast milk IgA supports the growth of *Bifidobacteria* spp. (127).

There is a complex interplay between the intestinal microbiome and the developing intestine. For example, commensal bacteria, including *Bifidobacterium* spp. and *Clostridium leptum* as well as *Faecealbacterium prausnitzii*, *Eubacterium rectale*, and *Roseburia* spp. produce short-chain fatty acids (SCFAs) (128–130). SCFAs such as butyrate, acetate, and propionate regulate inflammation (131–133). Specifically, butyrate inhibits LPS-induced inflammatory cytokines such as IL-1β, TNF-α, and IL-6 (134). Butyrate also enhances regulatory T-cell development and production of the anti-inflammatory cytokine IL-10 (135). In addition to producing SCFAs, these commensal bacteria occupy a niche in the intestine that prevents the overgrowth of potentially pathogenic bacteria. In preterm neonates, the growth of these harmful bacteria can have devastating consequences, and intestinal microbial dysbiosis has been repeatedly associated with the development of NEC (121–123, 136).

Numerous studies have investigated if increasing the abundance of commensal bacteria in the neonatal intestine with probiotics impacts the incidence of NEC. Although data point to a potential benefit of probiotics (137), this remains an area of controversy within the field of neonatology (138). There is a lack of consistency among probiotics used in clinical trials and the lack of regulation of available commercial products. Further research is needed before probiotics become a standard of care in preventing NEC.

## Milk composition by stage of lactation

Human milk composition by stage of lactation has been previously reviewed in detail (139–141). Colostrum is the first stage of milk production and consists of a high concentration of potentially beneficial and immunomodulatory components, including secretory IgA, lactoferrin, growth factors, cytokines, and HMOs (139, 141, 142). Although colostrum contains a high concentration of factors that are protective against NEC such as IgA (143), EGF (5) and HMOs (43), studies investigating provision of an extended course of exclusive colostrum feeding on the risk of NEC are limited by the volume of maternal colostrum available. Over the course of lactation, milk content shifts to a composition that promotes infant growth and development with higher concentrations of lactose and fat in mature milk relative to colostrum, although the composition is influenced by a variety of maternal factors (141).

## Donor milk

Donor milk is an alternative source of human milk feeds when maternal milk is not available in adequate quantities. The composition of donor milk is significantly impacted by pasteurization and storage (144–147), and it is generally derived from a pool of high-producing donors, which can also lead to significant differences in milk composition from maternal milk. Meta-analyses point to a reduced risk of NEC for donor milk feeds, although it remains to be determined if there is a significant impact on death or neurodevelopmental impairment (148). The Milk trial is a recently completed randomized control trial that will address these questions by investigating the impact of donor milk vs. formula on neurodevelopmental outcomes 22–26 months.

## Breast milk fortification and risk of NEC

The caloric density of human milk feeds is commonly increased with the addition of fortifiers to enhance the growth of preterm neonates. Comparison of human milk-based and bovine milk-based fortifiers has not demonstrated a significant difference in either mortality or morbidity, including in NEC rates, between these types of fortification (149, 150). This remains an area of active research.

## Models for studying the roles of nutrients in NEC

Due to the limited availability of human neonatal intestinal samples, mechanistic studies into the pathogenesis of NEC rely upon animal studies and *in vitro* models. NEC-like intestinal inflammation is induced in neonatal rats, mice, rabbits, and piglets through brief periods of hypoxia, feeding formula, LPS, and bacteria isolated from the microbiota of infants with NEC (151, 152). These models have been used to investigate the roles of prebiotics, probiotics, maternal milk constituents (milk proteins, HMOs), vitamins, fatty acid supplementation, and amino acids in the pathophysiology of NEC (81, 82, 91, 95, 153–155).

Numerous *in vitro* models and cell lines have been used in studies investigating the mechanisms involved in NEC (156–159). The human colorectal adenocarcinoma cell line, Caco-2, is often used to study intestinal disease; however, these cells are unable to differentiate into goblet cells leading to a lack of mucus secretion. The human colon adenocarcinoma cell line, HT-29, is also used to study NEC and will differentiate and produce mucus-secreting goblet cells in specific cell culture conditions (160). The benefit of using cell lines for mechanistic studies include abundance, reproducibility, and ease of culture. However, the cellular complexity of the intestine is hard to emulate in these static monoculture cell models. In addition, the relevance of findings in these adult tumor cell lines to neonatal disease is questionable. To overcome these difficulties, an *ex vivo* three-dimensional (3D) human organoid culture was developed to bridge the gap between traditional cell culture and studying primary human samples.

Gastrointestinal organoids are multicellular, 3D structures developed from primary intestinal stem cells (ISCs) or from inducible pluripotent stem cells (iPSCs) (161, 162). Intestinal organoids (also called enteroids) contain multiple intestinal epithelial cell types, which retain their critical structural and functional properties of the intestinal epithelium, such as barrier integrity, mucus and antimicrobial peptide (AMP) secretion, and differentiation capabilities. Therefore, enteroids allow for the study of numerous biologic properties, including barrier function, inflammation, cellular proliferation, therapeutic responses, nutrient effects, and epithelial-microbial interactions (163, 164). Limitations of using enteroids include their polarity and difficulties in co-culturing with immune and endothelial cells (165, 166). These challenges led to the development of novel Gut-on-a-Chip or Intestine-on-a-Chip platforms (167, 168).

The Gut-on-a-Chip platform is a technical advance on enteroid models due to the ability to co-culture multiple cell types, provide a constant flow of media, access the apical side of the epithelium, and mimic intestinal peristalsis via stretch (167). We recently developed a NEC-on-a-Chip model using enteroids cultured from intestinal tissue obtained from neonates undergoing intestinal surgery (168). These enteroids were cultured on a microfluidic device in the presence of an endothelial cell line and the intestinal microbiome of an infant that died from NEC (168). In these culture conditions, we detected cellular and gene expression changes similar to what is observed upon studying samples from neonates with NEC (168). This study highlights the scientific relevance of Gut-on-a-Chip models for mechanistic investigations related to the pathogenesis of NEC.

## Conclusions and future directions

The intestine of the preterm neonate faces the difficult task of meeting their nutritional requirements while still undergoing postnatal development and being inundated with microbes and the challenges posed by critical illness. Optimizing the provision of the beneficial components of breast milk is central to supporting neonates through this difficult stage. Disrupted intestinal homeostasis and dysregulated inflammation can lead to NEC. Breast milk provides protection against this dangerous disease, and further research into how modulation of enteral nutrition can prevent NEC and improve outcomes for neonates with NEC remains a priority.
